# Mitochondrial-targeted SS-31 peptide attenuates radiation-induced cardiomyocyte senescence

**DOI:** 10.1093/jrr/rrag048

**Published:** 2026-07-15

**Authors:** Lixin Xie, Jinzi Wu, Jiaojiao Fan, Kimberly J Krager, Nukhet Aykin-Burns, Songlin Li, Elisabet Børsheim, Xingyun Qi, Marjan Boerma, Huiliang Zhang

**Affiliations:** Department of Pharmacology and Toxicology, University of Arkansas for Medical Sciences, 4301 W. Markham Street, Little Rock, AR 72205, USA; Department of Medical Genetics & Molecular Biochemistry, Lewis Katz School of Medicine, Temple University, 3440 N Broad St, Kresge Hall, Philadelphia, PA 19140, USA; Department of Pharmacology and Toxicology, University of Arkansas for Medical Sciences, 4301 W. Markham Street, Little Rock, AR 72205, USA; Division of Radiation Health, University of Arkansas for Medical Sciences, 4301 West Markham Street, Little Rock, AR 72205, USA; Division of Radiation Health, University of Arkansas for Medical Sciences, 4301 West Markham Street, Little Rock, AR 72205, USA; Department of Medical Genetics & Molecular Biochemistry, Lewis Katz School of Medicine, Temple University, 3440 N Broad St, Kresge Hall, Philadelphia, PA 19140, USA; Department of Pediatrics and Department of Geriatrics, University of Arkansas for Medical Sciences, 4301 West Markham Street, Little Rock, AR 72205, USA; Department of Biology, Rutgers University–Camden, Joint Health Sciences Center, 201 South Broadway, Camden, NJ 08103, USA; Division of Radiation Health, University of Arkansas for Medical Sciences, 4301 West Markham Street, Little Rock, AR 72205, USA; Department of Medical Genetics & Molecular Biochemistry, Lewis Katz School of Medicine, Temple University, 3440 N Broad St, Kresge Hall, Philadelphia, PA 19140, USA

**Keywords:** ionizing radiation, cardiomyocyte, cell senescence, mitochondria, SS-31

## Abstract

Exposure to ionizing radiation, such as from radiation therapy or accidental radiation exposure can have adverse effects on the heart. While radiation injury in the heart may include cardiomyocyte senescence, there are no available strategies to prevent this phenomenon. This study evaluated the effects of the mitochondrial-targeted peptide SS-31 (elamipretide) on radiation-induced cell senescence in cardiomyoblast H9C2 cells and human induced pluripotent stem cell derived cardiomyocytes (hiPSC-CMs). Exposure to γ-radiation at doses of 2, 5, and 10 Gy inhibited H9C2 cell proliferation in a dose-dependent manner, and induced senescence-associated beta-galactosidase (SA-β-gal) staining, a gold standard of cell senescence. Treatment with SS-31 (1 μM) for 7 days reduced SA-β-gal positive staining from 67% to 38% in H9C2 cells under 10 Gy radiation. SS-31 also prevented increases in the expression of p16 and p21, two well-accepted senescence markers, in irradiated H9C2 cells and hiPSC-CMs. SS-31 decreased the canonical senescence-associated secretory phenotype markers TNF-α, IL-6, and IL-1β. SS-31 also reversed the BAX/bcl-2 ratio, a marker of mitochondrial-related apoptosis. Moreover, SS-31 mitigated mitochondrial production of reactive oxygen species. Interestingly, we found that a dose of 10 Gy increased mitochondrial respiration, and SS-31 reversed this elevation. This study suggests that SS-31 is a promising compound that may prevent radiation-induced cardiomyocyte senescence.

## INTRODUCTION

Radiotherapy is a widely used and effective treatment for cancer, especially in the intermediate and advanced stages. Approximately 50% of cancer patients undergo radiotherapy during the course of their treatment [1]. However, despite its therapeutic benefits, radiotherapy is associated with significant side effects. In addition, exposure to ionizing radiation (IR) from a nuclear event may cause injuries in multiple organ systems [2]. One of the serious complications of radiation exposure involving the chest is radiation-induced heart disease (RIHD) [3]. RIHD encompasses a spectrum of cardiovascular conditions, including pericarditis, cardiomyopathy, coronary artery disease, valvular heart disease, and conduction system abnormalities [3, 4]. Currently, there are no effective interventions available to prevent or mitigate RIHD.

Mechanistically, RIHD is driven by a combination of DNA damage, inflammation, oxidative stress, and cell death, among others [5]. A key contributor to this pathology is mitochondrial injury, which involves mitochondrial DNA damage, excessive reactive oxygen species (ROS) generation, impaired energy metabolism, disruption of mitochondrial dynamics, and mitochondrial membrane permeabilization [6, 7]. Additionally, IR activates multiple cell death pathways, including apoptosis, necroptosis, pyroptosis, and ferroptosis, further contributing to tissue damage in various organs [6].

Exposure to IR has been shown to induce cardiomyocyte senescence as detected using senescence-associated β-galactosidase (SA-β-gal) staining, a widely accepted marker of cell senescence [8]. The induction of senescence is of particular concern in cardiomyocytes, which are terminally differentiated and have limited regenerative capacity. The accumulation of senescent cardiomyocytes over time is believed to contribute to cardiac aging and the development of cardiovascular diseases [9]. However, the underlying mechanisms of radiation-induced cardiomyocyte senescence remain poorly understood, and currently, there are no effective therapies targeting this aspect of RIHD.

SS-31 (H-D-Arg-Dmt-Lys-Phe-NH2) is a mitochondria-targeted peptide that binds to cardiolipin, a phospholipid abundant in the inner mitochondrial membrane. By stabilizing the mitochondrial cristae structure, SS-31 reduces superoxide production and mitigates oxidative stress [10]. SS-31 protects against a broad spectrum of diseases and organ systems, including Alzheimer’s disease [11], diabetes-associated visual impairment [12], and ischemia–reperfusion injuries in the liver [13], kidney [14], and heart [15], as well as in age-related cardiac diastolic dysfunction [16]. In this study, we investigated radiation-induced cell senescence in cardiomyoblast H9C2 cells and human induced pluripotent stem cell derived cardiomyocytes (hiPSC-CMs). Furthermore, we evaluated the effects of SS-31 on radiation-induced cardiomyocyte senescence.

## MATERIALS AND METHODS

### H9C2 cell culture

Cardiomyoblast H9C2 cells (ATCC) were cultured in Dulbecco’s modified Eagle’s medium (DMEM) supplemented with 10% fetal bovine serum (Gibco, 16140071) and 1% penicillin–streptomycin. Cells were maintained at 37°C with 5% CO_2_ and passaged every 3 days. On Day 3, cells were harvested using 0.05% trypsin–ethylenediaminetetraacetic acid (trypsin–EDTA; Gibco, 25200–056) and centrifuged at 300 g for 3 min and resuspended in fresh medium. Cell counts were determined using a hemocytometer.

### hiPSC-CMs culture and differentiation

hiPSCs (Gladstone) were cultured with mTeSR™1 basal medium supplemented with 5× supplement (Stemcell Technologies, 85 850) at 37°C with 5% CO_2_. As previously reported [17, 18], cells were seeded in 12-well plates to initiate differentiation. Following overnight incubation, the mTeSR medium was replaced with mTeSR supplemented with 1 μM Chiron (set as day −1). On day 0, the medium was changed to RPMI 1640 with bovine serum albumin (BSA) and ascorbic acid (RBA) with 4 μM Chiron. On Day 2 the medium was changed to RBA with 2 μM WNT-C59, followed by RBA alone on Day 4. On Day 6, the medium was replaced with RPMI with B-27 supplemented with insulin. From this point forward, the RPMI/B-27 supplement medium was replaced every other day. Spontaneous contractions, indicative of cardiomyocyte differentiation, were typically observed from Day 8 onward, and the hiPSC-CMs were irradiated on Day 10.

### Radiation exposure and SS-31 treatment

H9C2 cells were seeded in 70 mm dishes overnight and were exposed to a single dose of 2, 5, or 10 Gy of γ-rays. hiPSC-CMs on 12-well plates were exposed to a single dose of 10 Gy. The radiation was delivered at a dose rate of 0.88 Gy/min using a cesium-137 source cabinet irradiator (Mark 1, Model 68A, JL Shepherd & Associates, San Fernando, CA). Radiation dosimetry was performed with Gafchromic film (DOSE-MAP, Ashland Specialty Ingredients, Wayne, NJ) and an ion chamber (Exradin A20, Standard Imaging, Middleton, WI) and electrometer (×4000, Standard Imaging) that are calibrated for γ-rays once a year. Sham-irradiated control cells were placed in the irradiator room for the same duration without radiation exposure. After (sham-) irradiation, cells were returned to the incubator with 5% CO_2_ at 37°C.

**Fig. 1:**
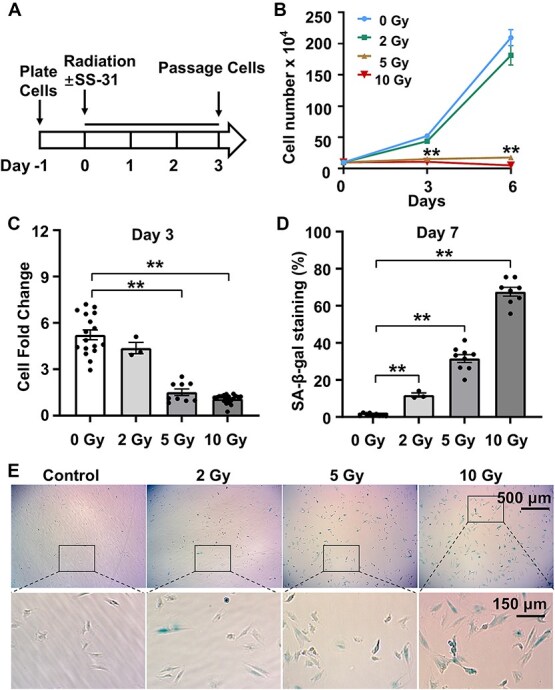
Radiation-induced cell senescence in H9C2 cells**.** (A) Schematic overview of the experiment protocol. H9C2 cells were plated on Day −1 and exposed to γ-rays on Day 0 in the presence or absence of SS-31. SS-31 was added 1 hour before irradiation. (B) Cell growth trace measured on day 3 and 6 post-irradiation, *n* = 3–19. (C) Fold change in cell number on day 3 post-irradiation compared to Day 0, *n* = 3–19. (D-E) Quantification and representative images of SA-β-gal staining on H9C2 cells 7 days after exposure to 0, 2, 5 and 10 Gy, *n* = 3–11. Scale bars: 500 μm in upper panels, 150 μm in lower enlarged panels. All data are presented as mean ± SEM. ^**^P < 0.01 vs control.

SS-31 (NovoPro) was added to the culture medium 1 hour before irradiation at 0.1, 1, 10, 30 μM, as outlined in Fig. 1A.

### Senescence associated β-galactosidase staining

Seven days after irradiation, cell senescence was assessed using a senescence-associated beta-galactosidase (SA-β-Gal) staining kit (CST, 9860). Cells were washed three times with phosphate-buffered saline (PBS) and fixed with 4% paraformaldehyde for 10 min. After two additional PBS washes, cells were incubated with SA-β-gal staining solution (pH = 6.0) at 37°C overnight. Following staining, cells were preserved with 70% glycerol. Images were acquired using a digital microscope camera (AmScope) and the β-Gal-positive cells were quantified using ImageJ software.

### Mitochondrial superoxide imaging

Seventy-two hours after irradiation, H9C2 cells were plated on a coverslip and incubated with 5 μM MitoSOX Red and 200 nM MitoTracker Green for 30 min at 37°C, then imaged by LSM 880 (ZEISS) confocal microscope. The ratio of MitoSOX Red (excitation 561 nm, emission >565 nm) to MitoTracker Green (excitation 488 nm, emission 505–530 nm) was used to determine the mitochondrial superoxide levels, as we previously reported [19].

### Flow cytometry cell cycle analysis

Forty-eight hours post-irradiation, cells were washed with ice-cold PBS and fixed overnight at 4°C in cold 70% ethanol. After fixation, cells were washed with cold PBS twice and incubated with 100 μl of 100 μg/ml RNase A (Thermo Scientific, EN0531) at 37°C for 30 min to degrade RNA. Then, the fixed cells were stained with 50 μg/ml propidium iodide (PI, Sigma, P4864) at 4°C for 30 min. The cell cycle was evaluated by flow cytometry (BD LSRFortessa) with excitation 535 nm and emission ˃ 617 nm and analyzed by FlowJo Software.

### Western blotting

Cells were collected on Days 2, 3, or 6 after irradiation, as indicated in each figure for western blotting. The cells were washed three times with PBS and lysed with radioimmunoprecipitation assay (RIPA) lysis buffer (ChemCruz, sc-24948A). The protein concentrations were determined using a BCA protein assay (Thermo, 23 225). After separation by sodium dodecyl sulfate–polyacrylamide gel electrophoresis (SDS-PAGE), proteins were transferred onto a polyvinylidene difluoride (PVDF) membranes (Fisher, IPVH00010). Membranes were blocked with 1% BSA or 1% non-fat milk in tris-buffered saline with Tween 20 (TBST) for 1 hour at room temperature, followed by overnight incubation at 4°C with the following primary antibodies: TNFα (MILLIPORE, AB1837P, 1:2000), IL-6 (Sigma, SAB5700632, 1:2000), p16 (Invitrogen, PA5–20379, 1:2000), p21 (Invitrogen, 14–6715-81, 1:2000), Bax (Invitrogen MA5–14003, 1:1000), Bcl-2 (Abcam, ab196495, 1:2000), Lamin B1 (CST, 13435, 1:2000), Caspase 7 (CST, 9492S, 1:2000), Caspase 8 (CST, 4790S, 1:2000), Caspase 9 (CST, 9508S, 1:1000), Cytochrome c (Medchemexpress, HY-P80102, 1:3000), VDAC1 (Medchemexpress, HY-P80369, 1:3000), OxPhos Rodent WB Antibody Cocktail (Invitrogen, 45–8099, 1:2000), or β-actin (Bio-techne, MAB8929, 1:5000). Horseradish peroxidase (HRP)-conjugated anti-rabbit IgG (MP Biomedicals, 0855689, 1:5000) and anti-mouse IgG (MP Biomedicals, 0855564, 1:5000) secondary antibodies were utilized. Detection substrate SuperSignal™ West Pico PLUS Chemiluminescent Substrate (ThermoFisher, 34 580) was added, and the membranes were imaged by FluorChem M (ProteinSimple). The bands were quantified by ImageJ software.

### XF96 seahorse respiration assay

Forty-eight hours after irradiation, the H9C2 cells were plated at 5000 cells/well in a Seahorse XFe96 plate (Agilent) and incubated at 37°C in 5% CO_2_ for 6 hours. The culture medium was then replaced with 100 μl of XF DMEM Medium (Agilent, 103 575–100) supplemented with 10 mM glucose, 1 mM pyruvate, and 2 mM glutamine. Plates were incubated for 1 hour at 37°C without CO_2_. Oxygen consumption rates were measured in a Seahorse XF96 Flux Analyzer. Mitochondrial stress assay compounds were injected sequentially as follows: oligomycin A (10 μM), carbonyl cyanide-p-trifluoromethoxyphenylhydrazone (FCCP) (10 μM), antimycin A (5 μM) plus rotenone (5 μM). Parameters including ATP production-related respiration, mitochondrial proton leak, and mitochondrial respiration control ratio (RCR) were calculated as previously described [20].

### Cell ATP level assay

Forty-eight hours after irradiation, cells were seeded into opaque 96-well plates at 5 × 10^3^ cells/well, with DMEM-only wells as background controls. After cells were attached, ATP level was assessed using the CellTiter-Glo® Luminescent Cell Viability Assay Kit (Promega, G7570). In brief, 100 μl of CellTiter-Glo® reagent was added to each well, mixed for 2 min, incubated at room temperature for 10 min, and luminescence was recorded.

### Mitochondrial and cytosolic fraction isolation and cytochrome c blotting

Cells were harvested 48 hours post-irradiation by trypsinization, washed with PBS, and maintained on ice, and all centrifugation steps were performed at 4°C. Cell pellets were resuspended in 1 ml mitochondrial isolation buffer (MIB, in mM, 300 sucrose, 10 HEPES, 0.2 EDTA) and homogenized using a Teflon homogenizer. Homogenates were centrifuged at 800 g for 10 min to remove nuclei and debris. The supernatants were centrifuged at 8000 g for 15 min to pellet mitochondria. The supernatant was collected as the cytosolic fraction and further purified by two additional centrifugations at 8000 g for 15 min. The mitochondrial pellet was washed twice in MIB with 8000 g centrifugation for 15 min, then 100 μl lysis buffer was added and protein concentration was determined by BCA assay. To quantify Cytochrome c levels, Western blot analysis was performed. Protein expression was normalized using VDAC1 as the loading control for the mitochondrial fraction and β-actin for the cytosolic fraction. The primary antibodies used were Cytochrome c (MedChemExpress, HY-P80102, 1:3000), VDAC1 (MedChemExpress, HY-P80369, 1:3000), and β-actin (Bio-Techne, MAB8929, 1:5000). The secondary antibodies and imaging condition are described in Section 2.7.

### Flow cytometric analysis of apoptosis

Forty-eight hours post-irradiation, cells were harvested, washed twice with ice-cold PBS, and resuspended in Annexin V binding buffer at a density of 1.5 × 10^5^ cells per 100 μl. Apoptosis was quantified using the FITC Annexin V Apoptosis Detection Kit I (BD Biosciences, 556 547) according to the manufacture’s instructions. Briefly, 100 μl of cell suspension was incubated with 5 μl of FITC Annexin V and 5 μl of PI for 15 min at room temperature in the dark. Following the addition of 400 μl of binding buffer, samples were analyzed by flow cytometry (BD LSRFortessa) within 1 hour. Unstained, Annexin V-only, and PI-only stained controls were utilized for compensation and quadrant gating.

### Statistical analysis

All experiments were repeated at least three times. All data are presented as mean ± SEM. The data were analyzed by one-way analysis of variance (ANOVA) followed by Dunnett post hoc test or two-tailed Student’s *t-*test with GraphPad software. A *P*-value of <0.05 was considered statistically significant.

## RESULTS

### Radiation dose-dependently induces H9C2 cell senescence

To investigate radiation-induced cell senescence, we exposed H9C2 cells to γ-rays at 2, 5, and 10 Gy. Following irradiation, cells were passaged every three days, and cell numbers were counted to assess proliferation rates (Fig. 1A). A 2 Gy dose resulted in only a modest reduction in cell growth by Day 3 and Day 6 post-irradiation (Fig. 1B and C). In contrast, 5 Gy significantly slowed proliferation, while 10 Gy completely suppressed cell growth by Day 3 (Fig. 1B and C). A higher dose of 15 Gy induced rapid cell death within 24 hours post-irradiation (data not shown).

To evaluate cell senescence, SA-β-gal staining, a gold standard of cell senescence [21], was performed on Day 7 post-irradiation. In addition to a minor effect on cell proliferation, 2 Gy induced senescence in ~12% of cells (11.8 ± 1.1%) (Fig. 1D and E). Exposure to 5 Gy nearly tripled the percentage of senescent cells compared with that in the 2 Gy group to 31.5 ± 2.1% (Fig. 1D and E), and 10 Gy further increased this to 67.5% ± 2.4% (Fig. 1D and E). Collectively, these results demonstrate that IR induces H9C2 cell senescence in a dose-dependent manner.

### SS-31 protects H9C2 cells from radiation-induced cell senescence

**Fig. 2:**
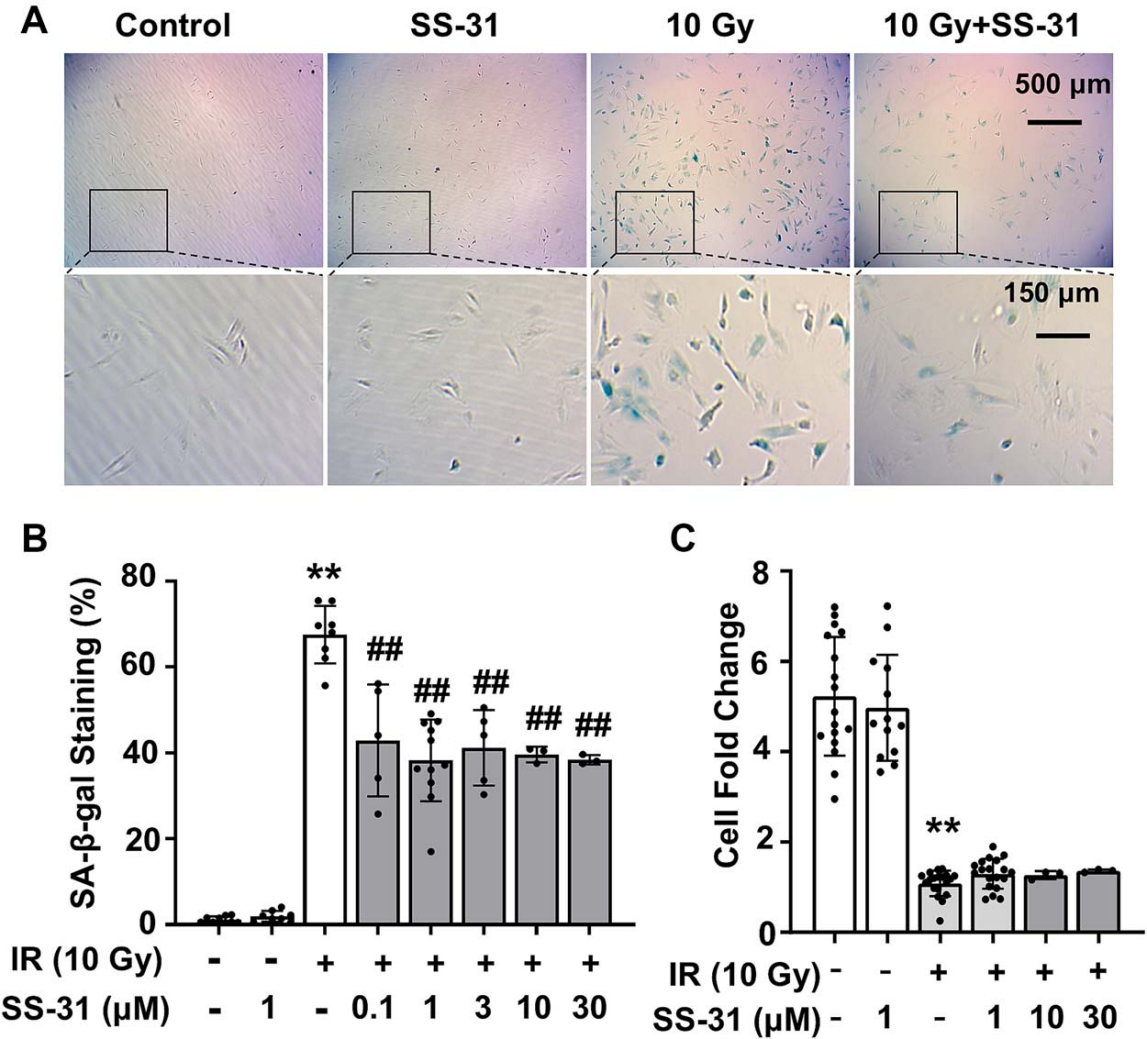
SS-31 attenuates cell senescence in H9C2 cells exposed to 10 Gy. (A) Representative images of SA-β-gal staining in H9C2 cells 7 days after 10 Gy, with or without SS-31 at 1 μM. Scale bar: 500 μm in the upper panel and 150 μm in the lower enlarged panel. (B) Quantification of SA-β-gal positive cells 7 days post-irradiation, *n* = 3–11. (C) Fold change in cell number 3 days post-irradiation compared to day 0, *n* = 3–19. All data are presented as mean ± SEM. ^**^P < 0.01 vs control; ##*P* < 0.01 vs irradiation.

Given that a dose of 10 Gy induced senescence in the majority of H9C2 cells, this dose was selected for subsequent experiments to evaluate the effects and underlying mechanisms of the mitochondrial targeted peptide SS-31. SS-31 was added to the medium 1 hour prior to irradiation at concentrations ranging from 0.1 to 30 μM. Treatment with 1 μM SS-31 significantly reduced the percentage of SA-β-gal positive cells from 67% to 38% following a dose of 10 Gy (Fig. 2A). We further tested the dose effect of SS-31 and found that even at 0.1 μM, SS-31 markedly reduced radiation-induced cell senescence. However, increasing the concentration to 3, 10, or 30 μM did not yield further reductions in percentage of senescent cells (Fig. 2B). Based on this, 1 μM SS-31 was used in subsequent mechanistic studies. Cell proliferation was minimal in the irradiated cultures, with or without SS-31 (Fig. 2C). These findings suggest that while SS-31 attenuates radiation-induced cell senescence in H9C2 cells, it does not promote cell proliferation. This is further supported by the cell cycle arrest test, see Section 3.3.

### S‌S-31 fails to reverse the radiation-induced cell cycle arrest

**Fig. 3:**
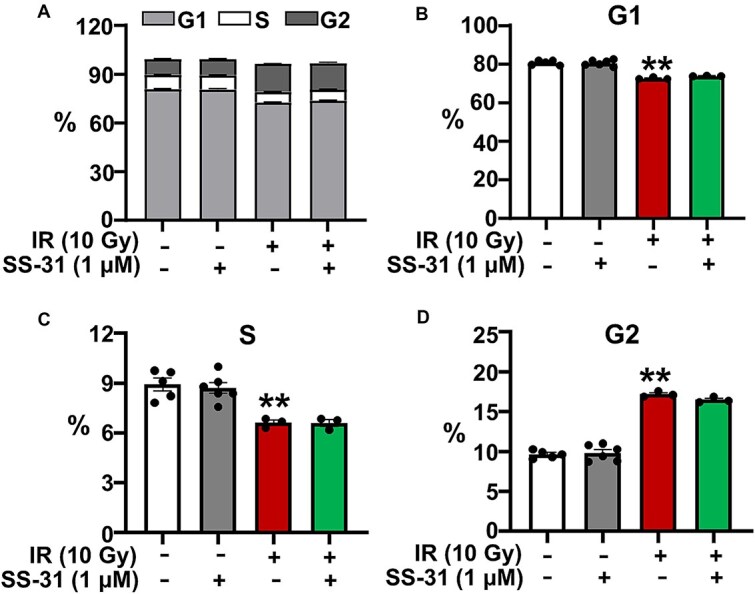
SS-31 has no effect on the radiation-induced cell cycle arrest in H9C2 cells. Flow cytometric analysis of the cell cycle using propidium iodide (PI) staining 48 hours after irradiation. Quantification of the percentage of cells in the G1 (B), S (C) and G2 (D) phases. *n* = 5 in each group. All data are presented as mean ± SEM. ^**^P < 0.01 vs control.

To determine whether SS-31 influences radiation-induced cell cycle arrest, H9C2 cells were analyzed by PI staining followed by flow cytometry (Figs S1 and 3A). Exposure to 10 Gy significantly decreased the fractions of cells in the G1 phase from 80.7 ± 0.5% to 72.6 ± 0.5% (Fig. 3B) and S phases from 8.9 ± 0.4% to 6.6 ± 0.2% (Fig. 3C). Correspondingly, the fraction of cells in G2 phase increased from 9.7 ± 0.2% to 17.2 ± 0.2% (Fig. 3D), reflecting impaired cell cycle progression. Therefore, in line with the earlier findings that SS-31 does not restore cell proliferation after exposure to γ-rays (Fig. 2C), treatment with SS-31 has no significant effect on the radiation-induced alteration in cell cycle distribution (Fig. 3A–D).

### SS-31 reduces radiation-induced senescence-related protein markers

**Fig. 4:**
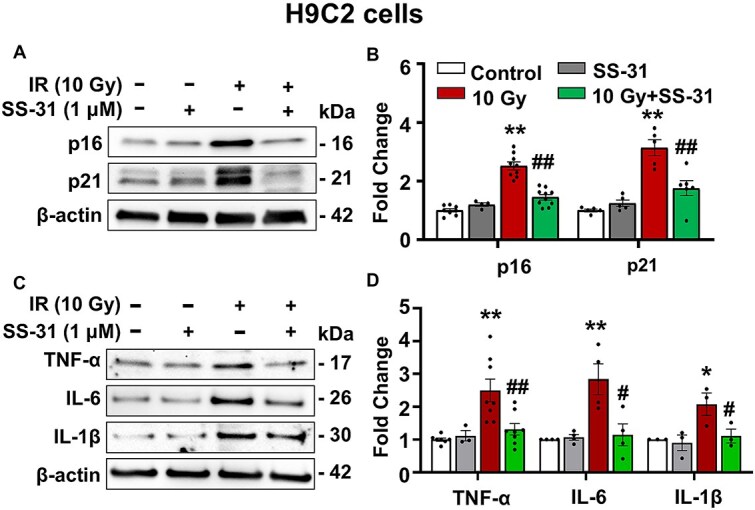
SS-31 decreases the expression of radiation-induced cell senescence markers in H9C2 cells**.** (A, C) Representative western blot images showing the expression of senescence markers p16 and p21 (A); senescence-associated secretory phenotype (SASP) markers TNF-α, IL-6 and IL-1β (C). (B, D) Quantification of corresponding protein expression levels, relative to control, *n* = 3–10. All data are presented as mean ± SEM. ^*^P < 0.05 vs control; ^**^P < 0.01 vs control; #*P* < 0.05 vs irradiation; ##*P* < 0.01 vs irradiation.

To further investigate the anti-senescent effects of SS-31, we examined its impact on key senescence-associated protein markers following exposure to γ-rays. Treatment with 1 μM SS-31 prevented the radiation-induced up-regulation of cyclin-dependent kinase suppressors p16 and p21 at 48 hours (Fig. 4A and B). Additionally, SS-31 attenuated the radiation-induced upregulation of senescence-associated secretory phenotype (SASP) proteins, including TNFα, IL-6, and IL-1β, after 72 hours of treatment (Fig. 4C and D). However, SS-31 has no protection on the radiation-induced decrease in Lamin B1 (Fig. S2).

**Fig. 5:**
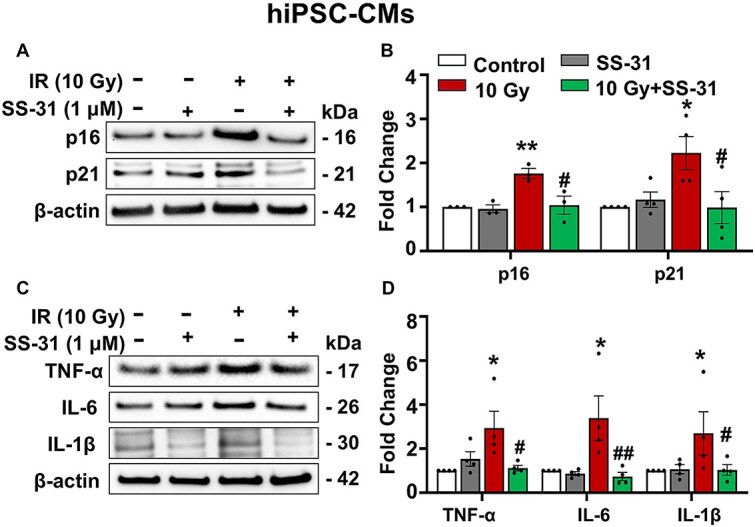
SS-31 decreases the expression of radiation-induced senescence markers in hiPSC-CMs**.** (A, C) representative western blot images showing the expression of senescence markers p16 and p21 (A); senescence-associated secretory phenotype (SASP) markers TNF-α, IL-6 and IL-1β (C). (B, D) quantification of corresponding protein expression levels, *n* = 3–10. All data are presented as mean ± SEM. ^*^P < 0.05 vs control; ^**^P < 0.01 vs control; #*P* < 0.05 vs irradiation; ##*P* < 0.01 vs irradiation.

As H9C2 cell is a cell line, to assess the translational relevance of these findings, we extended this experiment to hiPSC-CMs. Similar to the findings in H9C2 cells, 10 Gy γ-rays induced marked increases in p16 and p21 protein levels in hiPSC-CMs at day 7, which were significantly reduced by SS-31 treatment (Fig. 5A and B). Furthermore, SS-31 pretreatment decreased the radiation-induced elevations of SASP markers TNFα, IL-6, and IL-1β (Fig. 5C and D). In summary, these findings confirm that SS-31 prevents radiation-induced senescence in human cardiomyocytes.

### SS-31 reduces radiation-induced mitochondrial-related apoptosis protein level

**Fig. 6:**
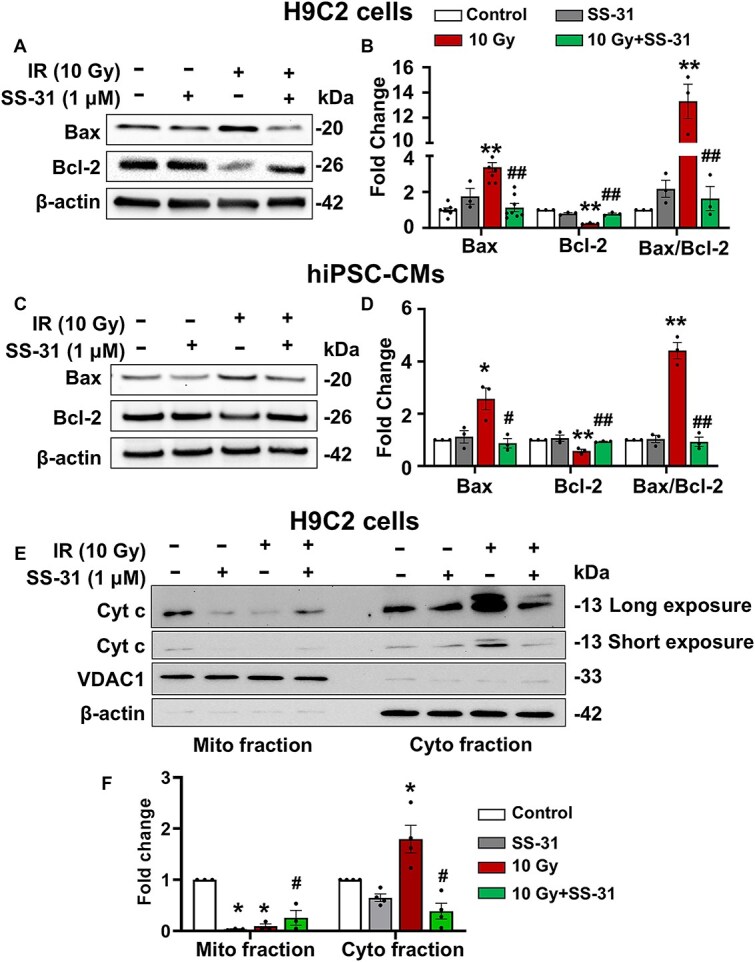
SS-31 reduces radiation-induced mitochondrial-related apoptosis proteins in H9C2 cells and hiPSC-CMs**.** (A, C) Representative western blot images showing the expression of mitochondrial apoptosis-related proteins Bax and Bcl-2 in H9C2 cells (A) and hiPSC-CMs (C). (B, D) Quantification of corresponding protein expression levels, n = 3–10. Experimental groups include control, SS-31 treatment, 10 Gy radiation, and 10 Gy radiation plus SS-31 treatment, as indicated in the figure. (E) Representative western blot images of cytochrome c (Cyt c) distribution. Protein levels were analyzed in isolated mitochondrial and cytosolic fractions. (F) Quantitative analysis of Cyt c expression. Cyt c levels were normalized to VDAC1 for the mitochondrial fraction and β-actin for the cytosolic fraction. *n* = 3–4. All data are presented as mean ± SEM. ^*^P < 0.05 vs control; ^**^P < 0.01 vs control; #*P* < 0.05 vs irradiation; ##*P* < 0.01 vs irradiation.

Mitochondrial dysfunction is often a leading cause of cellular senescence. Therefore, we examined mitochondrial-dependent cell apoptosis. In H9C2 cells, a dose of 10 Gy induced the expression of the pro-apoptotic protein BAX and decreased levels of the anti-apoptotic protein Bcl-2 (Fig. 6A and B). Treatment with 1 μM SS-31 effectively restored BAX and Bcl-2 expression to levels comparable to those in control cells (Fig. 6A and B). Similarly, in hiPSC-CMs, 1 μM SS-31 completely prevented alterations in mitochondrial-related apoptosis markers of BAX, Bcl-2 and the BAX/Bcl-2 ratio induced by 10 Gy (Fig. 6C and D). 10 Gy irradiation significantly increased the translocation of Cytochrome c (Cyt c) from the mitochondria to cytosol and SS-31 decreased the Cyt c release (Fig. 6E and F), while the total Cyt c expression remained unchanged (Fig. S3). Notably, 10 Gy irradiation did not increase the expression of apoptosis proteins, including caspase-7, −8 and − 9 (Fig. S4A and B), and SS-31 had no further effect on these levels (Fig. S4A and B). The absence of 10 Gy radiation induced apoptosis was further confirmed by Annexin V and PI dual staining and flow cytometry assay (Fig. S4C). Collectively, these results demonstrate that 10 Gy radiation induces mitochondrial-associated apoptotic protein level change without triggering apoptosis. Furthermore, SS-31 protects cardiomyocytes from radiation-induced mitochondrial-associated apoptotic protein level change and Cyt c release to cytosol.

### SS-31 attenuates radiation-induced mitochondrial ROS production

**Fig. 7:**
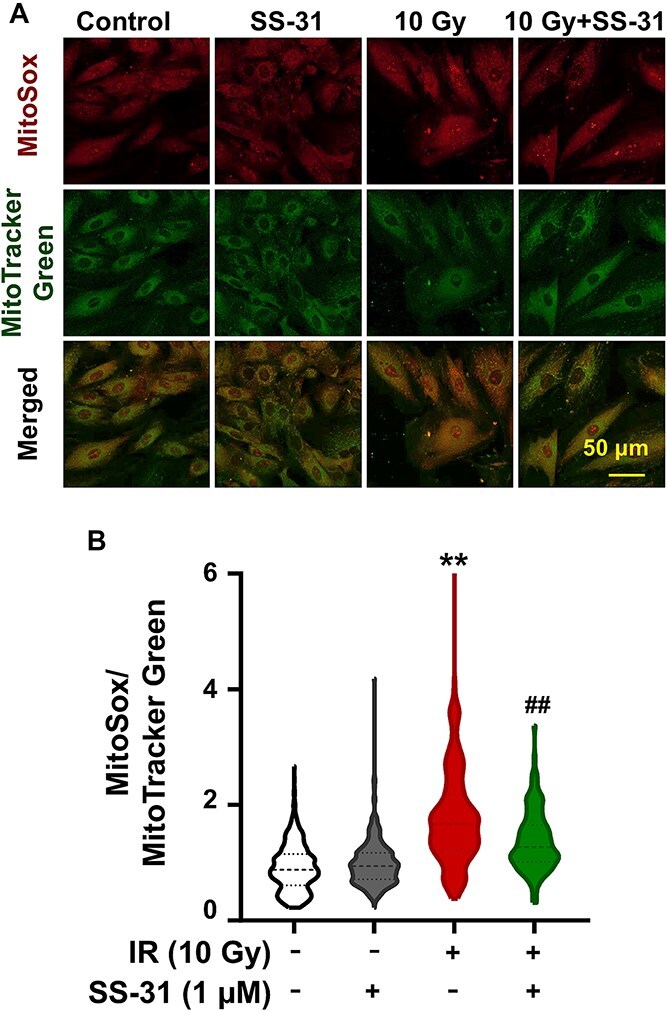
SS-31 reduces radiation-induced mitochondrial reactive oxygen species (ROS) production in H9C2 cells. (A) Representative confocal microscopy images showing mitochondrial superoxide levels. Upper panels: Mitochondrial superoxide indicator MitoSOX; middle panels: Mitochondrial mass indicator MitoTracker green; lower panels: Merged images of MitoSOX and MitoTrackerGreen. Scale bar: 50 μm. (B) Quantification of the mitochondrial superoxide level expressed as the ratio of MitoSOX to MitoTrackerGreen fluorescence. *n* = 127–466 cells from three independent experiments. All data are presented as mean ± SEM. ^**^P < 0.01 vs control; ##*P* < 0.01 vs irradiation.

SS-31 is known to localize to the mitochondrial inner membrane [10] and to reduce the mitochondrial ROS production in the aged cardiomyocyte [19]. To evaluate whether SS-31 suppresses radiation-induced mitochondrial ROS production, mitochondrial superoxide levels were assessed using the ratio of mitochondrial superoxide indicator mitoSOX to the mitochondrial mass indicator MitoTrackerGreen [19]. Exposure to 10 Gy resulted in more than a two-fold increase in mitochondrial superoxide production compared with control cells (Fig. 7A, B). Treatment with 1 μM SS-31 effectively reduced this radiation-induced ROS increase, restoring mitochondrial superoxide production levels to those of the control group (Fig. 7A, B). These data suggest that SS-31 ameliorates irradiation-induced oxidative stress in H9C2 cells by reducing mitochondrial ROS production.

### SS-31 normalizes the radiation-induced elevation of mitochondrial respiration

**Fig. 8:**
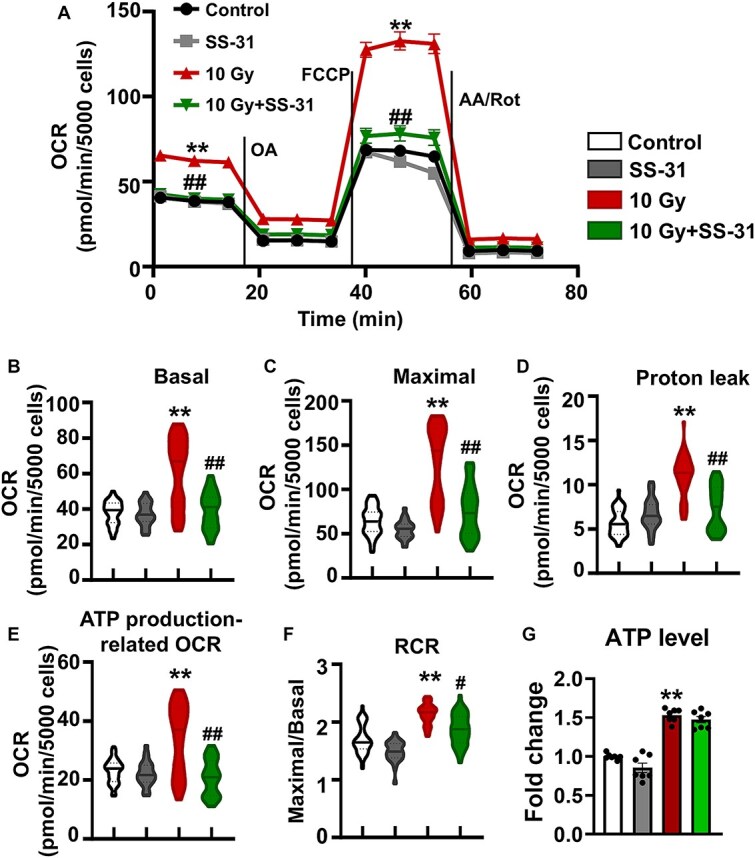
SS-31 reverses the radiation-induced elevation of mitochondrial respiration in H9C2 cells. (A) Average trace of oxygen consumption rate (OCR) measured by seahorse XF96 mitochondrial stress assay in intact H9C2 cells. OA: Oligomycin A (10 μM); FCCP: Carbonyl cyanide-p-trifluoromethoxyphenylhydrazone (10 μM); AA/rot: Antimycin A (5 μM) plus rotenone (5 μM). (B-F) Quantification of basal respiration (B), maximal respiration (C), proton leak (D, oxygen consumption rate after oligomycin minus the oxygen consumption rate after AA/Rot), ATP production-related respiration (E), and respiration control ratio (RCR) (F). Data represented as mean ± SEM from *n* = 7 independent experiments. (G) Quantification of total cellular ATP levels. Data were normalized to control cells to represent relative changes in ATP content. Data represented as mean ± SEM. *n* = 7 tests from 3 independent biological preparations. ^**^P < 0.01 vs control; ##*P* < 0.01 vs irradiation.

Given that SS-31 targets the mitochondrial inner membrane and decreases radiation-induced mitochondrial ROS production in H9C2 cells (Fig. 7), we next investigated the effects of radiation and SS-31 on mitochondrial respiration. Unexpectedly, exposure to 10 Gy markedly increased both the baseline oxygen consumption rate (basal respiration) and the highest level of oxygen consumption rate (maximal respiration) (Fig. 8A–C). About 10 Gy also elevated mitochondrial proton leak (Fig. 8D), reflected by oxygen consumed by protons re-entering the mitochondria without generating ATP. This increased proton leak indicates reduced mitochondrial efficiency, a phenotype previously reported in aged cardiomyocytes [19]. Moreover, 10 Gy enhanced the ATP production-related respiration (Fig. 8E). To determine if the observed increase in ATP-linked respiration correlated with actual metabolic output, we quantified total cellular ATP content. We found that 10 Gy irradiation significantly elevated ATP levels (Fig. 8G), suggesting a compensatory hypermetabolic response to radiation-induced stress. 10 Gy also increased mitochondrial RCR (the ratio of maximal to the basal respiration, Fig. 8F). To explore the mechanism of this induction of mitochondrial respiration, we checked the protein levels of mitochondrial oxidative phosphorylation complexes and found mild increases in complex I, III and V, accompanied by a reduction in complex IV following 10 Gy (Fig. S5).

Treatment with 1 μM SS-31 for 48 hours completely normalized the radiation-induced increases in mitochondrial respiration to levels comparable to controls (Fig. 8). SS-31 administered 1 hour before irradiation significantly reduced basal and maximal respiration (Fig. 8B, C), as well as the excessive mitochondrial proton leak (Fig. 8D), ATP production-related respiration (Fig. 8E) and RCR (Fig. 8F). Note that, although SS-31 treatment reduced respiration linked to ATP production, it did not significantly alter the total cellular ATP levels in irradiated cells (Fig. 8G). This discrepancy suggests that SS-31 enhances mitochondrial coupling efficiency, maintaining high energetic output despite lower oxygen consumption. On the other hand, SS-31 treatment exerted minor effects on respiratory complex proteins and prevented only the reduction in the expression of complex IV (Fig. S5A, E) and further increased the complex III (Fig. S5A, D). Taken together, these results demonstrate that SS-31 directly modulates mitochondrial function in irradiated cardiomyocytes, normalizing aberrant mitochondrial respiration and reducing proton leak, and these effects may be due to direct interaction with the mitochondrial inner membrane, rather than respiratory complex expression.

## DISCUSSION

This study examined radiation-induced cell senescence in cardiomyocytes, an understudied aspect of RIHD. We provide multiple lines of evidence of cardiomyocyte senescence following radiation exposure. First, consistent with a previous report [8], radiation dose-dependently increased SA-β-gal staining in H9C2 cells, a golden standard of the cell senescence. Second, radiation exposure led to cell growth retardation, another characteristic feature of senescent cells [22], which may be attributed to cell cycle arrest. Third, the elevation of senescence markers p16 and p21, along with the upregulation of SASP markers, including TNFα, IL-6, and IL-1β [22], further confirmed radiation-induced senescence in both H9C2 cells and hiPSC-CMs. Notably, upregulation of p16 and p21 in senescence is observed not only in proliferating cells but also in terminally differentiated non-dividing cells, such as primary cardiomyocytes, which have lost regenerative capacity [23]. The SASP, characterized by the secretion of cytokines, chemokines, growth factors, and proteases, represents another hallmark of senescent cells and contributes to tissue inflammation and dysfunction [24]. Fourth, radiation induced mitochondrial ROS production, a well-recognized hallmark of cell senescence [22]. Elevated mitochondrial ROS promotes oxidative stress, damages mitochondrial and cellular components, and disrupts redox homeostasis, thereby accelerating age-related cellular decline. Furthermore, oxidative modifications of contractile proteins may impair cardiac function [25]. Finally, radiation-induced cell senescence was accompanied by increased mitochondrial proton leak, a phenotype also observed in intact aged cardiomyocytes [19] and in mitochondria isolated from aged cardiac tissue [26]. Taken together, these findings demonstrate that the radiation-induced H9C2 cell senescence model effectively recapitulates key molecular and functional hallmarks of cellular aging. This model provides a robust and feasible platform for investigating the mechanisms underlying radiation induced cardiomyocyte dysfunction and for screening potential therapeutic agents that protect against radiation-induced cardiac cell senescence.

Our major finding, however, is that SS-31 prevents radiation-induced cellular senescence phenotypes without altering radiation-induced cell cycle arrest or growth inhibition. It is well established that radiation induces excessive ROS production in the heart [27, 28]. Elevated mitochondrial ROS further amplifies oxidative stress through a process known as ROS-induced ROS release [29, 30]. This oxidative stress contributes to mitochondrial and nuclear DNA damage [31], dysregulation of signaling transduction pathways, Ca^2+^ mishandling [32], and overall cellular dysfunction, ultimately accelerating senescence and increasing the risk of age-related diseases [25]. SS-31 incorporates into cardiolipin, a phospholipid abundant in the mitochondrial inner membrane, thereby stabilizing mitochondrial cristae structure and reducing excessive ROS production [10]. In this study, we demonstrate that SS-31 prevents radiation-induced mitochondrial ROS overproduction, which represents a key mechanism underlying its ability to prevent cell senescence. Moreover, while SS-31 effectively mitigates the transition into a full senescent phenotype, likely by preserving mitochondrial bioenergetics, it does not necessarily re-initiate the cell cycle in cells already impacted by radiation-induced DNA damage. These cells may remain in a state of stable cell-cycle arrest but maintain a more robust metabolic and energetic profile compared to their senescent counterparts.

An intriguing finding from this study is that radiation increased mitochondrial respiration in H9C2 cells. This is in contrast to radiation-induced decreases in mitochondrial basal respiration observed in the rat heart at 2 weeks after a single dose of 21 Gy [7, 33]. The elevation in basal and maximal respiration may partially compensate for the radiation-induced rise in mitochondrial proton leak. The excessive proton leak imposes an energetic burden on mitochondria, reducing respiration efficiency and enhancing the electron leakage, which in turn exacerbates superoxide generation [34]. From this perspective, attenuation of excessive mitochondrial proton leak may represent another mechanism by which SS-31 prevents radiation-induced cell senescence. More interestingly, increased proton leak is typically related to reducing the mitochondrial ability to produce ATP; however, we found that radiation elevates ATP production related oxygen consumption and increases mitochondrial RCR. Similar observations were reported *in vivo*, where mitochondrial ATP related respiration in salivary glands increased 24 hours after irradiation but returned to the levels in untreated groups by 48 hours [35]. Consistent with these findings, radiation was shown to elevate the ATP levels in EMT6 cells at 24 hours after exposure [36]. This enhancement of mitochondrial bioenergetic function may represent a compensatory response to meet the elevated energy demand required for the synthesis of senescence related proteins, especially the SASP proteins. Our data reveal a notable bioenergetic shift following SS-31 treatment in irradiated H9C2 cells. While radiation-induced stress initially triggers a compensatory increase in ATP production, this is accompanied by inefficient oxygen utilization and elevated mitochondrial ROS. The addition of SS-31 appears to stabilize this energetic state; however, it does so with a significantly reduced oxygen consumption rate (Fig. 8). Our data revealed that radiation increases the protein levels of electron transport chain complexes I, II, III and V, while decreasing complex IV expression. The complex II protein levels decreased in the rat heart *in vivo* at 2 weeks, 10 weeks, and 6 months after a single dose of 21 Gy [37]. This discrepancy may come from differences between the *in vitro* and *in vivo* radiation models or the post-irradiation time points. While radiation triggered a compensatory increase in Complex III expression, this effect was further synergistically augmented by SS-31 treatment. Together, the unchanged ATP level despite lower oxygen consumption is consistent with improved mitochondrial coupling efficiency, suggesting that SS-31 may facilitate a more efficient electron transport process. By stabilizing cardiolipin and optimizing electron transport chain supercomplex assembly, SS-31 likely enhances the efficiency of oxidative phosphorylation. This allows the cell to maintain homeostatic ATP yields with lower oxygen requirements, effectively increasing the P/O ratio and mitigating the metabolic ‘wastage’ typically observed in irradiated, dysfunctional mitochondria. Further investigation is warranted to elucidate the molecular basis of radiation-induced mitochondrial respiratory activation.

Several limitations should be acknowledged in this study. First, although we have extended our experiments from rat cardiomyoblast H9C2 cells to human hiPSC-CMs, further validation of the protective effect of SS-31 *in vivo* will provide stronger translational relevance. Second, RIHD may involve endothelial injury and subsequent myocardial ischemia [3], and there is some evidence of radiation-induced senescence in endothelial cells [38]. In this study, we focused exclusively on cardiomyocytes; therefore, it would be valuable to determine whether SS-31 confers protective effects in other cardiac cell types.

In summary, we established a radiation-induced cardiomyocyte senescence model suitable for screening potential therapeutic agents to prevent this aspect of RIHD. Using this research model, we identified the mitochondrial targeted small peptide SS-31 as a promising candidate that protects against radiation-induced cardiomyocyte senescence. The protective effects of SS-31 appear to be mediated, at least in part, by reducing excessive mitochondrial ROS production and attenuating mitochondrial proton leak.

## Supplementary Material

Supplemental_1_rrag048

Supplemental_2_rrag048

Supplemental_3_rrag048

Supplemental_4_rrag048

Supplemental_5_rrag048

## Data Availability

The original data generated and analyzed in the study are included within the article and its supplementary materials. Further inquiries can be directed to the corresponding author.
