# Supplementary figures and images for: Mitochondrial-targeted SS-31 peptide attenuates radiation-induced cardiomyocyte senescence
Source: J Radiat Res. 2026 Jul 15;67(4):525–37. doi: 10.1093/jrr/rrag048 (PMC13400571; doi:10.1093/jrr/rrag048)

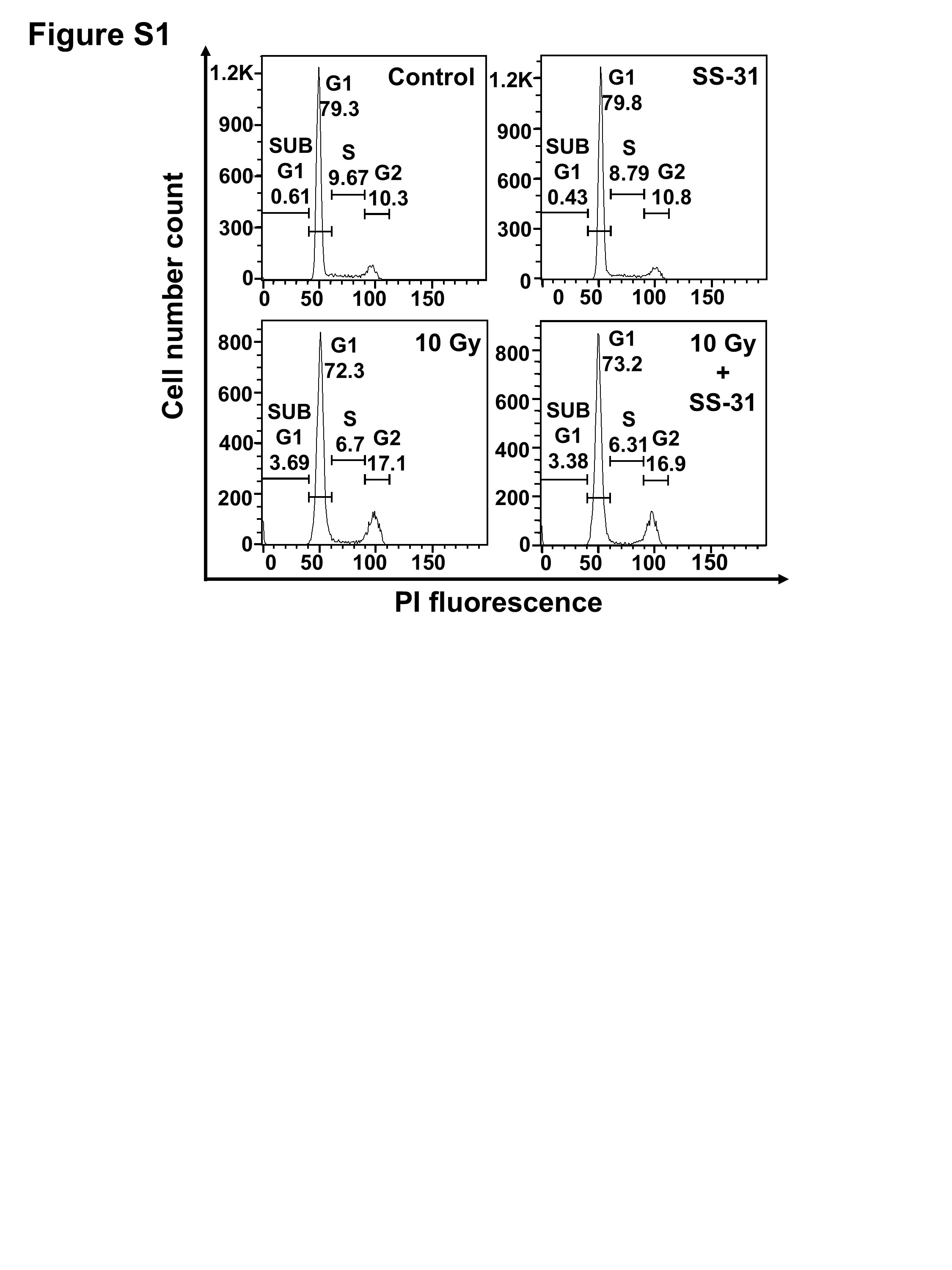

Supplement: Supplemental_1_rrag048 [file supplemental_1_rrag048.jpeg]

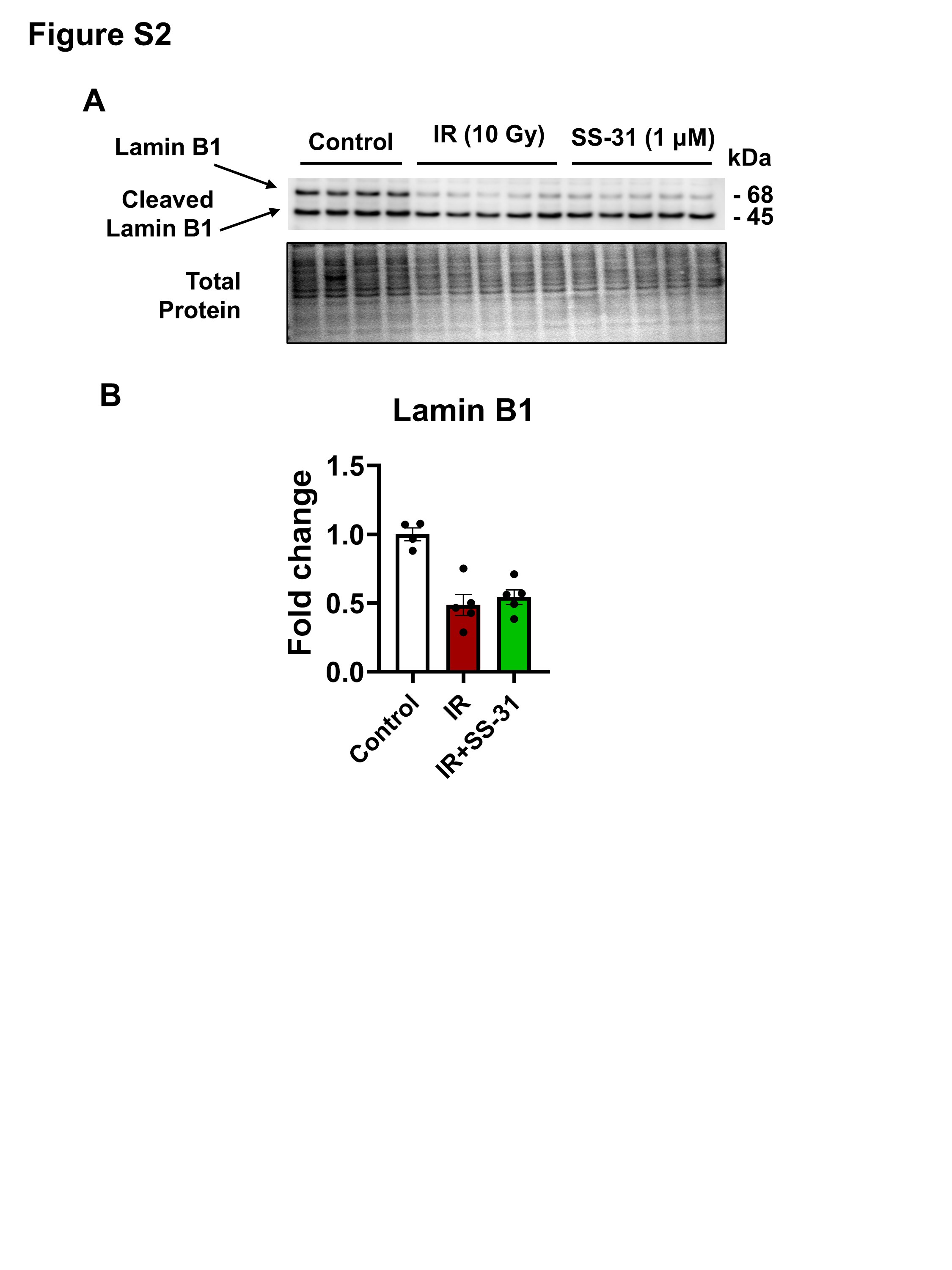

Supplement: Supplemental_2_rrag048 [file supplemental_2_rrag048.jpeg]

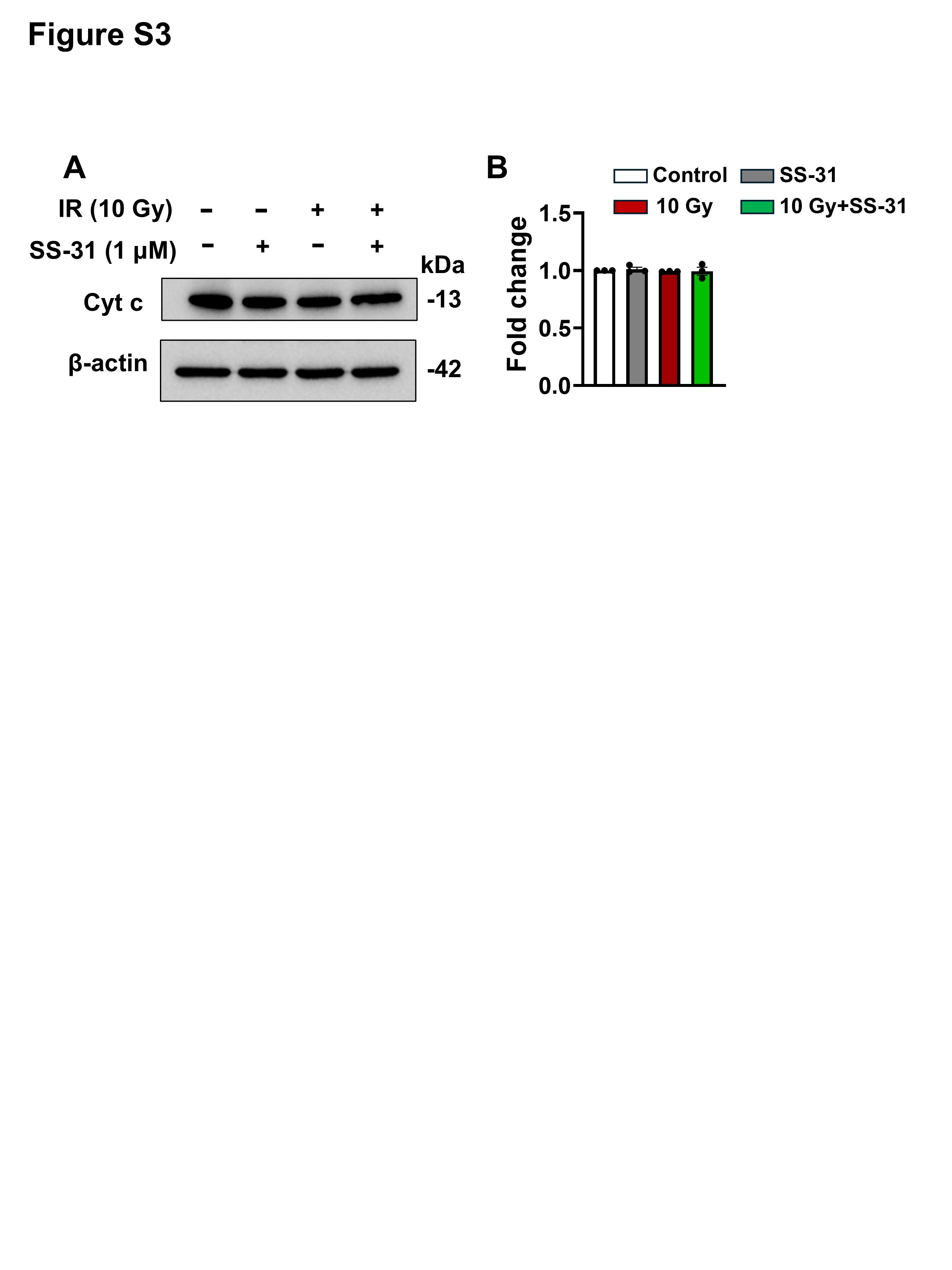

Supplement: Supplemental_3_rrag048 [file supplemental_3_rrag048.jpeg]

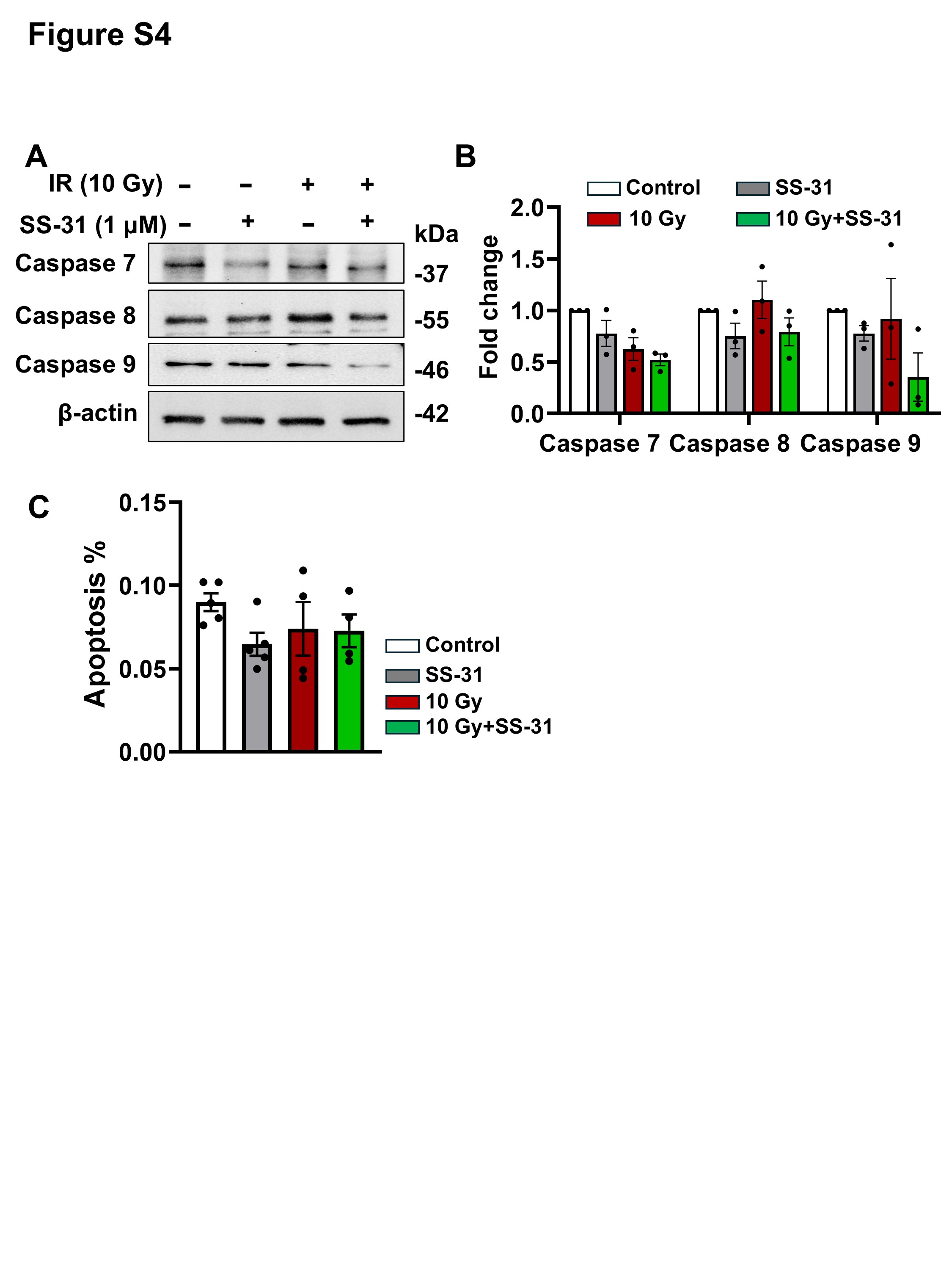

Supplement: Supplemental_4_rrag048 [file supplemental_4_rrag048.jpeg]

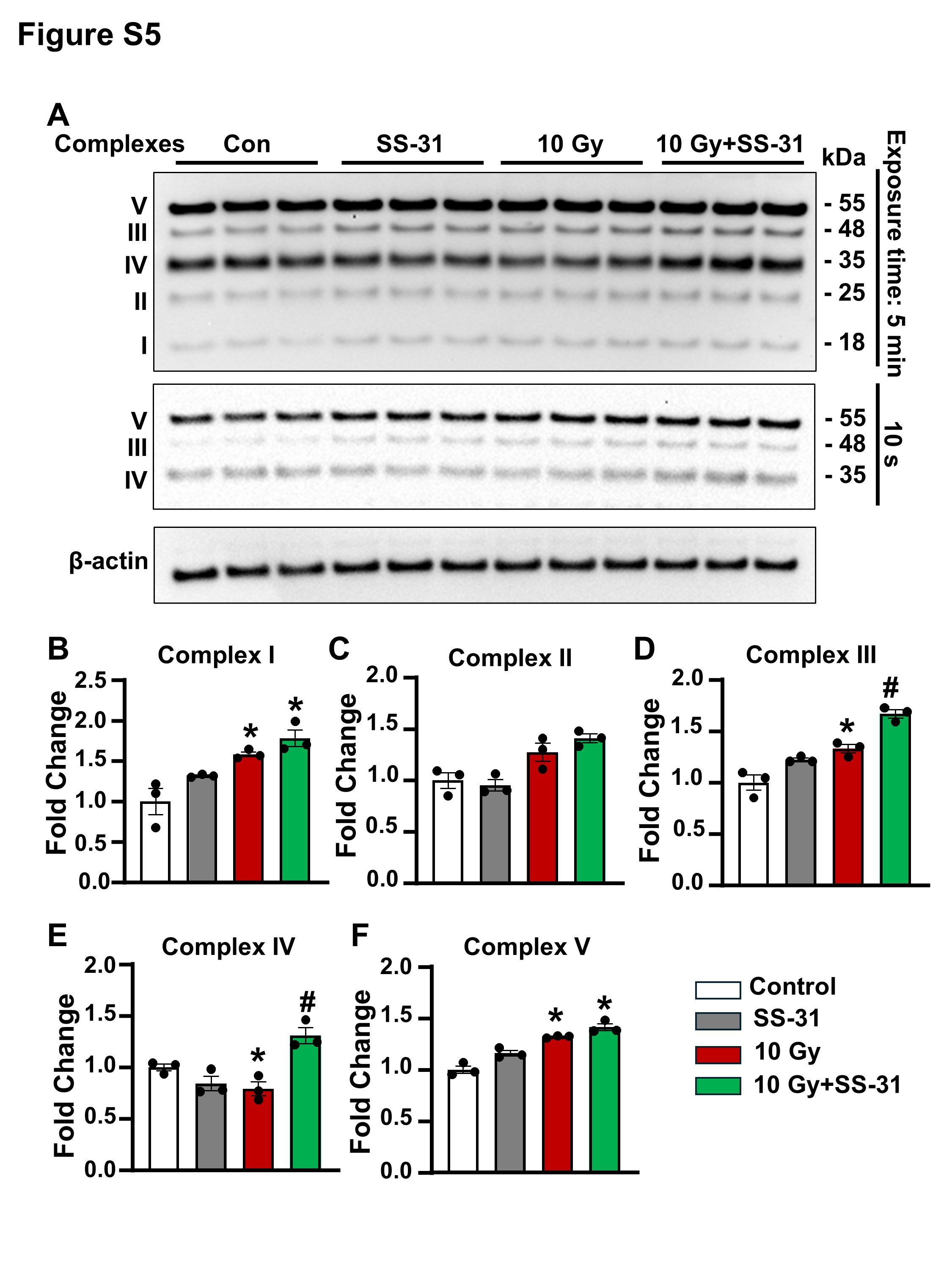

Supplement: Supplemental_5_rrag048 [file supplemental_5_rrag048.jpeg]
